# Spinal Arachnoid Cysts: A Single-Center Preliminary Surgical Experience with a Rare and Challenging Disease

**DOI:** 10.3390/jpm15060234

**Published:** 2025-06-05

**Authors:** Alessio Iacoangeli, Love Chibuzor Ilochonwu, Giulia Mazzanti, Gabriele Polonara, Lauredana Ercolani, Alessandra Marini, Michele Luzi, Roberto Trignani, Stefano Bruni, Edoardo Barboni, Maurizio Gladi, Maurizio Iacoangeli, Denis Aiudi

**Affiliations:** 1 Neurosurgery Clinic, Marche Polytechnic University, Azienda Ospedaliero Universitaria (AOU) delle Marche, 60126 Ancona, Italy; lovekic94@gmail.com (L.C.I.); giulia.mazzanti89@gmail.com (G.M.); mauriziogladi@gmail.com (M.G.); neurotra@gmail.com (M.I.); denis.aiudi@gmail.com (D.A.); 2 Neurosurgery Division, Azienda Ospedaliero Universitaria (AOU) delle Marche, 60126 Ancona, Italy; alessandra_marini@ospedaliriuniti.marche.it (A.M.); michele.luzi@ospedaliriuniti.marche.it (M.L.); roberto.trignani@ospedaliriuniti.marche.it (R.T.); 3 Department of Neuroradiology, Marche Polytechnic University, Azienda Ospedaliero Universitaria (AOU) delle Marche, 60126 Ancona, Italy; gabriele.polonara@ospedaliriuniti.marche.it; 4 Department of Neurorehabilitation, Marche Polytechnic University, Azienda Ospedaliero Universitaria (AOU) delle Marche, 60126 Ancona, Italy; lauredana.ercolani@ospedaliriuniti.marche.it; 5 Interventional Neuroradiology, Azienda Ospedaliero Universitaria (AOU) delle Marche, 60126 Ancona, Italy; stefano.bruni@ospedaliriuniti.marche.it; 6 Neuroanesthesia Unit, Azienda Ospedaliero Universitaria (AOU) delle Marche, 60126 Ancona, Italy; edoardo.barboni@ospedaliriuniti.marche.it

**Keywords:** arachnoid cyst, arachnoiditis, adhesiolysis, arachnoidolysis, intramedullary cyst, intradural cyst

## Abstract

**Background:** Spinal arachnoid cyst development (SAC) is a rare and debilitating disease with a non-well-defined treatment strategy: a series of five patients diagnosed with SAC and submitted to neurosurgical treatment was retrospectively analyzed. **Objectives**: SACs represent 1–2% of all spinal neoplasms; they can be extradural, intradural, or intramedullary, with intradural arachnoid cysts (IDACs) comprising only 10% of these cases. The rarity of SACs and the lack of consensus on the best treatment strategies represent a care challenge: the aim of this study is to explore the effectiveness and outcomes of the neurosurgical management in patients with SACs treated at our institution. **Methods**: Adult patients who underwent surgical treatment for SACs between January 2020 and December 2023 were included in the study: clinical onset, imaging, surgical technique, and neurological long-term status were retrospectively analyzed. **Results**: Five patients (three males, two females; average age 53.4 years) were included. The most common symptoms described were paresthesia, gait disturbances, and back pain. Radiological imaging indicated that most cysts were at the thoracic level. Surgical interventions primarily involved cyst resection and adhesiolysis. Post-operative outcomes showed overall improvement or stability in Karnofsky Performance Status (KPS) and American Spinal Injury Association Impairment Scale (ASIA) scores in the majority of cases, although complications and recurrences occurred. **Conclusions**: Surgical resection combined with adhesiolysis may prevent the worsening of neurological impairment and potentially improve pain control and clinical outcomes in patients with SACs. However, careful and tailored management is required due to the high potential of complications and recurrences.

## 1. Introduction

Spinal arachnoid cysts (SACs) are rare lesions, accounting for 1–2% of all spinal tumors [1]. The majority are in the extradural space (EDACs), while only 10% of all arachnoid cysts are intradural (IDACs) [2]. SACs can occur in both pediatric and adult patients, most commonly presenting between the fifth and sixth decades of life, with significant variation in their distribution among genders [3]. In most cases, spinal cysts are considered idiopathic, as no underlying cause can be identified. However, SACs may sometimes develop secondary to an arachnoiditis phenomenon caused by spinal trauma, inflammation, infection, or as a result of medical and surgical interventions [4]. These cysts are most frequently located at the thoracic spine, although they can potentially develop at any level from extradural to intradural or intramedullary [5,6,7]. Since they are space-occupying lesions with a compressive effect on the spinal column, SACs can cause neurological dysfunction and myelopathy, often requiring surgical intervention; therapeutic management becomes particularly challenging when arachnoiditis is involved. The rarity of these cases limits a comprehensive understanding of their underlying mechanisms, resulting in a lack of consensus on the optimal treatment strategy. This study aims to explore the effectiveness and outcomes of the neurosurgical management in patients with SACs treated at our institution.

## 2. Materials and Methods

Adult patients who underwent surgical treatment at our Neurosurgery Department for SACs between January 2020 and December 2023 were included in this observational, single-center, retrospective study: clinical onset, imaging, surgical technique, and neurological long-term status were analyzed. The applied inclusion and exclusion criteria are listed in Table 1.

The study was conducted in accordance with the Declaration of Helsinki, and ethical approval was waived by our local ethics committee in view of the retrospective nature of the study and all the procedures being performed were part of the routine care.

### Data Collection

Patient demographics, medical history (e.g., previous spinal trauma, infections, or surgeries), clinical onset, pre- and post-operative neurological evaluations (Karnofsky Performance Status—KPS—and American Spinal Injury Association Impairment Scale-ASIA-scores), surgical details, and post-operative complications were obtained from medical records and outpatient reports. Radiological imaging, including mandatory pre- and post-operative MRIs, was collected to assess lesions’ reduction or resolution, recurrence, and the occurrence of surgical complications: the longitudinal and transverse diameters of the SAC were measured on T2-weighted sagittal MRI images. In all cases, the diagnosis was confirmed through histological examination after intraoperative biopsy.

## 3. Results

### 3.1. Patients Overview

Our retrospective series, based on the application of inclusion and exclusion criteria, was composed of five patients. The age of the patients at the time of diagnosis ranged from 43 to 68 years, with an average age of 53.4 years. The group consisted of three male and two female patients. Etiology was distributed as follows: the most common (60%) was post-operative (three patients had undergone prior spinal surgeries), 20% was post-traumatic (one patient had suffered a burst fracture of L1 treated with lumbar arthrodesis) and another 20% was infectious (one patient had a history of an epidural abscess). The most common symptoms described were paresthesia, gait disturbances, and back pain; in addition, neurogenic bowel or bladder (*n* = 2), limb weakness (*n* = 2), paraplegia (*n* = 1), and sexual dysfunction (*n* = 1) occurred (see Table 2).

### 3.2. Radiological Data

In most cases, the lesion was located at the thoracic level (*n* = 3); one case was at the cervical spine and another one at the thoracolumbar junction. In all patients, the pathology involved more than one vertebral level, ranging from two to four segments. Among these cysts, three were intradural and two were intramedullary.

### 3.3. Surgical Details and Post-Operative Outcomes

The majority of patients underwent surgical resection of the cystic formation combined with adhesiolysis (*n* = 4), while in one patient, the procedure included opening the syrinx cavity following lysis of adherences. The symptoms that most frequently improved after surgery were limb weakness (*n* = 3), gait ataxia (*n* = 2), and neurogenic bladder (*n* = 2). In contrast, only one patient each experienced improvement in paresthesia or dysesthesia and lower back pain. The average pre-operative and post-operative KPS scores were 70% and 82%, respectively, reflecting an overall improvement in health status in all patients except one. The ASIA level remained unchanged from pre-operative to post-operative in all cases except for two patients, whose ASIA level improved (from B to C and from C pre-operatively to D post-operatively). Surgical complications included cerebrospinal fluid fistulas (*n* = 2), which were resolved surgically, and one infectious complication. Additionally, four patients out of five experienced disease recurrence, necessitating further surgical re-interventions (see Table 2 and Table 3).

### 3.4. Illustrative Cases

#### 3.4.1. Case 1

**Figure 1:**
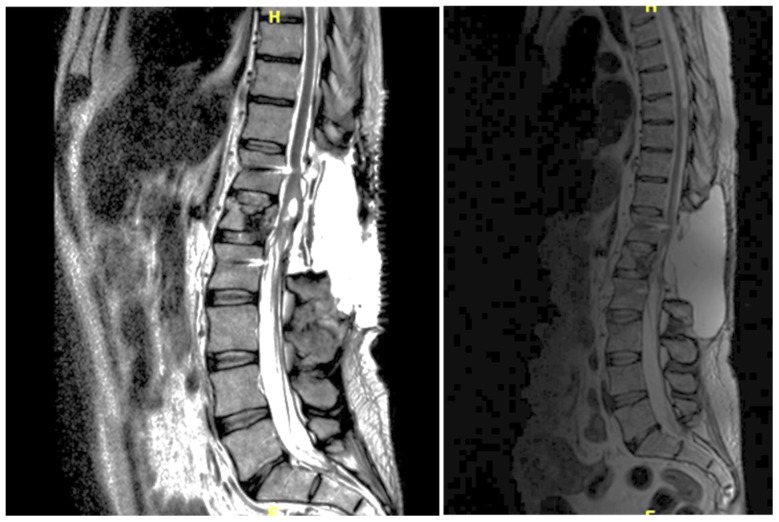
Case 1: pre-operative MRI (**left**) and post-operative MRI (**right**).

A 46-year-old man with a history of post-traumatic burst fracture of L1 in a car accident, treated with lumbar fusion at D12–L2, developed seven months later lower limb weakness, sensory loss, and bladder and bowel dysfunction (Figure 1). MRI revealed syringomyelia at D12–L2 compressing the conus medullaris. Initial surgery involved syrinx decompression through myelotomy, leading to significant symptom improvement; the ASIA score improved from C pre-operatively to D post-operatively. However, three months later, his symptoms recurred with an increased syrinx size. A second surgery introduced a syringo-subarachnoid shunt and removed scar adhesions, with temporary improvement, although a post-operative cerebrospinal fluid fistula occurred. He underwent a third surgery with a syringoperitoneal shunt, resolving the syrinx. Post-operatively, a 1-month follow-up MRI showed a significant reduction in the syrinx size and improvement in myelopathy: the patient’s neurological status improved, with gradual recovery of bladder and bowel functions.

#### 3.4.2. Case 2

**Figure 2:**
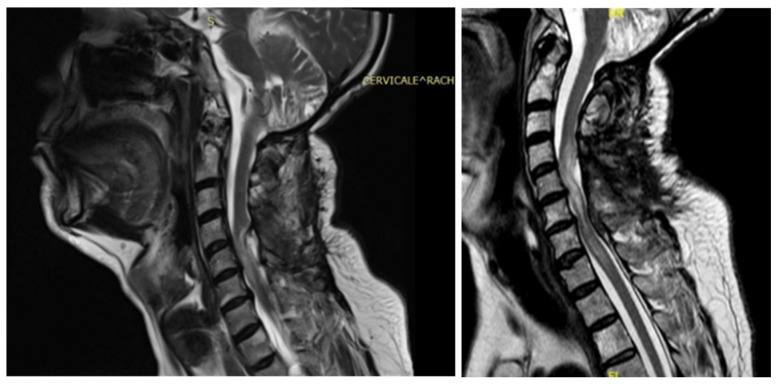
Case 2: pre-operative MRI (**left**) and post-operative MRI (**right**).

**Figure 3:**
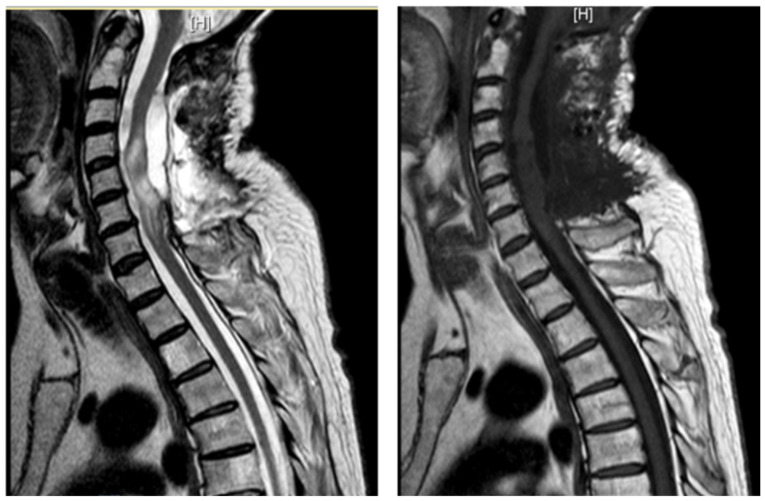
Case 2: pre- and post-operative MRI showing reduction in the myelopathy area.

A 48-year-old man with a history of multiple neurosurgical interventions since his childhood presented with left upper limb weakness and gait worsening seven months after the removal of a cervical spinal catheter previously inserted (Torkildsen shunt). Over the next five months, his condition deteriorated, resulting in an inability to walk independently. Neurological examination revealed left upper limb weakness and spastic paraparesis in the lower limbs. The MRI of the cervicodorsal spine showed myelopathy at the C5–C6 level with a dorsal cystic lesion (Figure 2). Surgery was performed to remove the cyst and perform the lysis of arachnoid adherence. A left-sided hemilaminectomy (C5–C7) exposed and excised the cyst compressing the spinal cord. Post-operatively, the patient initially improved but later developed tetraparesis. The MRI performed two months later showed persistent cord swelling and myelopathy extending to D1-D2 (Figure 3). A second surgery included decompressive laminectomy (C6–C7) and removal of intradural adhesions. Despite these interventions, the patient’s ASIA score B remained unmodified with persistent tetraparesis and only a slight upper limb improvement. Follow-up MRI revealed ongoing signal changes from C3–C4 to D4–D5. A third surgery revealed intraoperatively new arachnoid adhesions and included an exploratory biopsy. Despite no further neurological decline, MRI continued to show persistent intramedullary signal abnormalities at three-year follow-up.

#### 3.4.3. Case 3

**Figure 4:**
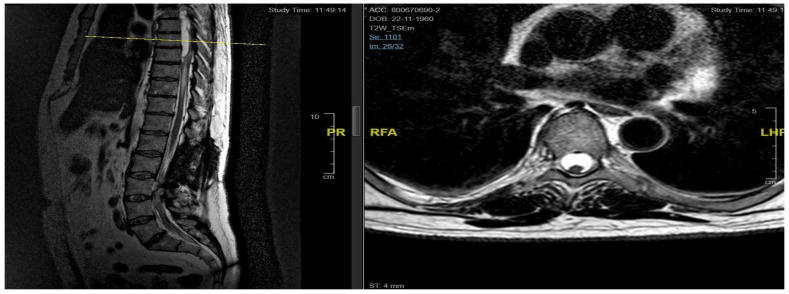
Case 3: pre-operative MRI showing an intradural and extramedullary cyst: pre-operative T2 sagittal view (**left**) and pre-operative T2 axial view (**right**).

**Figure 5:**
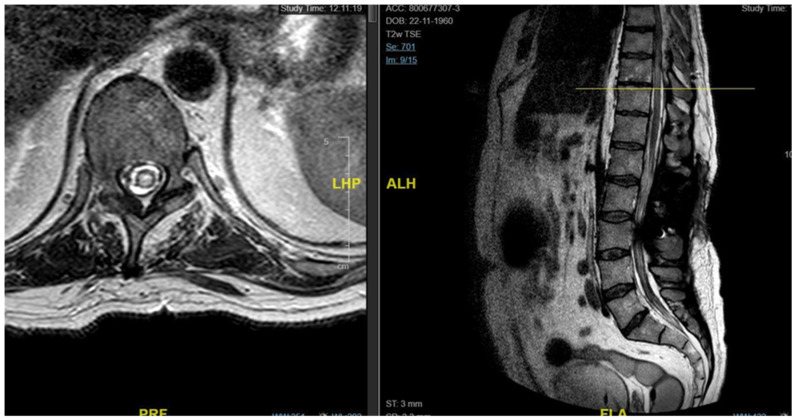
Case 3: post-operative MRI showing the absence of a recurrent cystic formation: post-operative T2 axial view (**left**) and post-operative T2 sagittal view (**right**).

A 62-year-old man, 10 months after surgery for the removal of a spinal cavernoma, developed recurrent left-sided lumbosciatica, paresthesia, hypoesthesia, and an episode of acute urinary retention. Neurological examination revealed weakness in the left lower limb, mild hyperreflexia, and neurogenic bladder. MRI showed an intradural-extramedullary cyst from D3 to D8 and an extradural component from D9 to D11, compressing the spinal cord and causing myelopathy (Figure 4). The patient underwent a left hemilaminectomy (D6–D10) for cyst removal. Histology confirmed a spinal arachnoid cyst. Post-operatively, the patient’s neurological status changed from ASIA B to C, and the one-week post-operative MRI showed the absence of the anterior cystic collection at the dorsal spine, with only a slight reduction in the area of myelopathy and persistent central spinal cord signal alteration at the D9–10 level (Figure 5). Eight months later, he experienced a worsening of symptoms, including low back pain and paraplegia. The performed MRI revealed a cystic mass from D4 to D8. He underwent a decompressive laminectomy (D5–D10) and cyst fenestration. Post-operative MRI showed resolution of the cyst but an extension of the myelopathic area. Despite these issues, the patient did not experience new neurological deficits over 3 years of follow-up.

#### 3.4.4. Case 4

**Figure 6:**
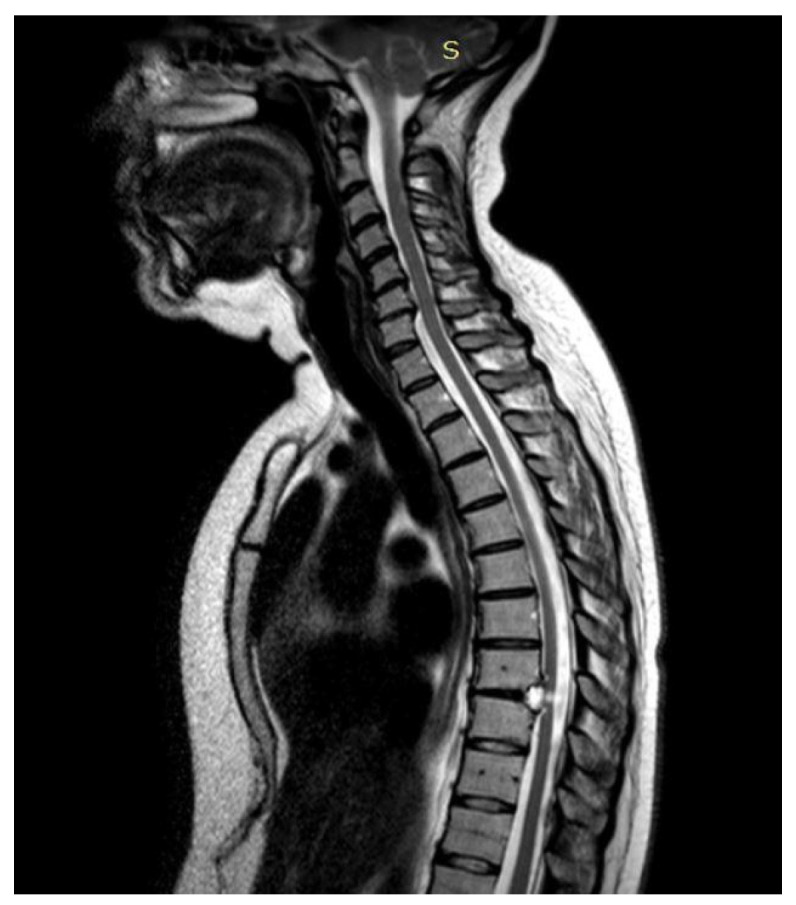
Case 4: pre-operative MRI illustrating a ventral intradural cyst.

A 43-year-old woman, following a microdiscectomy at D8–D9 and 2 years of symptom relief, developed sensory deficits and gait difficulties. Neurological evaluation revealed right lower limb weakness and an ataxic gait. Pre-operative MRI identified an intradural cyst at D8–D9 (Figure 6). Surgical intervention involved a right hemilaminectomy (D7–D9) to remove the cyst and resolve arachnoid adhesions. Histology confirmed an arachnoid cyst. An initial post-operative improvement was followed by symptoms worsening, including new onset of syringomyelia.

**Figure 7:**
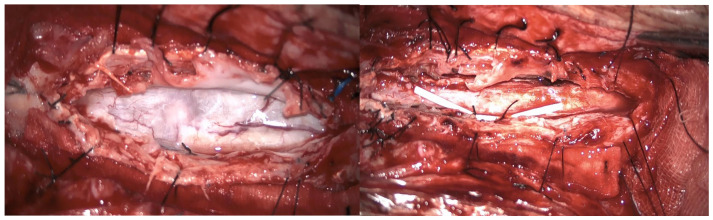
Case 4: syringoperitoneal shunt placement and adhesion lysis: scar and arachnoid adhesions(**left**); adhesion lysis and syringoperitoneal catheter draining the syringomyelic cavity (**right**).

**Figure 8:**
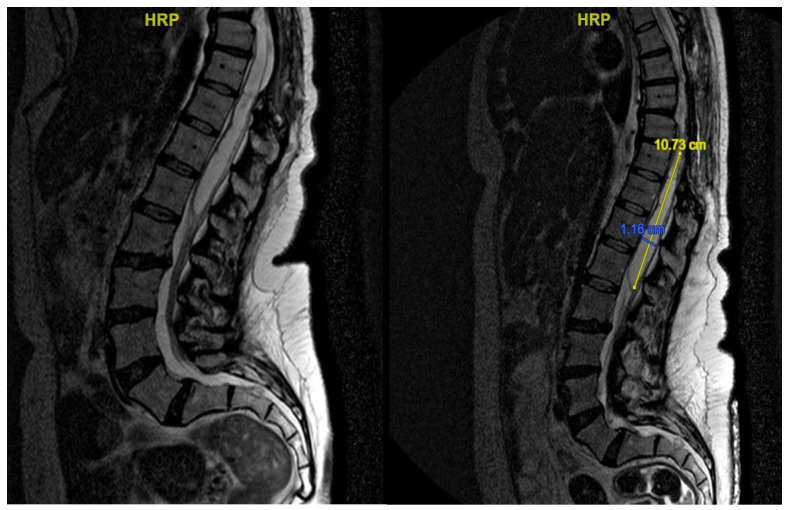
Case 4: post-operative MRI showing the reduction in syringomielic size: pre-operative (**left**) and post-operative (**right**).

A second surgery addressed the syringomyelia with laminectomy (D7–D10) and adhesion lysis: post-operative MRI showed resolution of the cyst but persistent malacic changes. A cerebrospinal fluid fistula was treated surgically. After further deterioration and expansion of the syringomyelic cavity, a third surgery, including reopening of the previous surgical incision and exposure of the laminectomy, allowed the dissection of numerous scar septa both above and below the dural plane, freeing the spinal cord circumferentially. A myelotomy was performed to drain the syringomyelic cavity and establish communication with the subdural space, placing a syringoperitoneal shunt and additional adhesion lysis (Figure 7). Post-operative 1-year MRI indicated reduced syringomyelic size (Figure 8). The patient did not experience a neurological worsening, with a stable ASIA score of C, after 4 years of follow-up.

#### 3.4.5. Case 5

A 68-year-old woman presented with progressively worsening bilateral lower limb hypoesthesia, leading to a neurosurgical evaluation about one year after symptom onset. Her medical history included an epidural abscess extending from D12 to L5, treated three years prior with targeted antibiotic therapy and CT-guided drainage.

**Figure 9:**
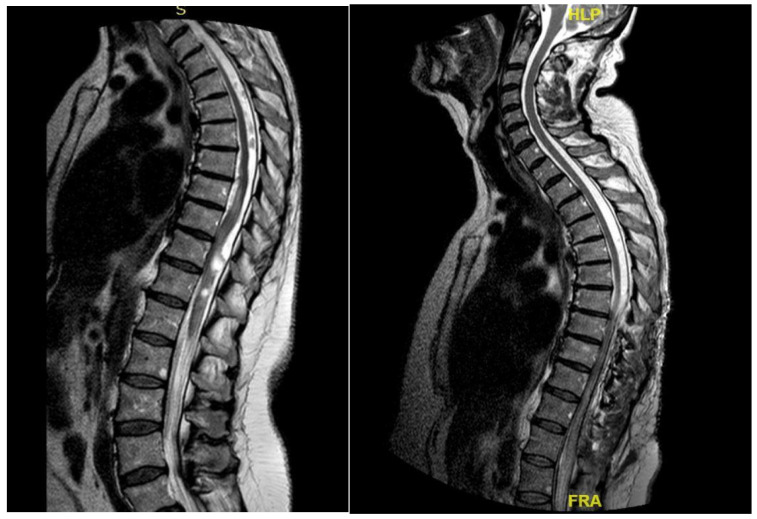
Case 5: pre-operative MRI (**left**) and post-operative MRI (**right**).

Neurological examination revealed ataxic gait and diffuse proprioceptive hypoesthesia in the lower limbs. MRI of the thoracolumbar spine with contrast identified intradural cystic collections at D8–D11, located dorsal to the spinal cord and causing signs of myelopathy (Figure 9).

The patient underwent decompressive laminectomy using piezosurgery at the affected spinal levels to remove the cysts and release arachnoid adhesions. Intraoperative monitoring of sensory and motor evoked potentials was performed. After exposing the dural plane, a full laminectomy was carried out, revealing the cystic lesion enveloping the spinal cord and numerous septa anchoring to neural structures, obstructing normal cerebrospinal fluid flow. The cyst was fenestrated into the subarachnoid space, and the adherent perimedullary structures were dissected to restore cerebrospinal fluid circulation and initial pulsatility of the exposed neural structures. Post-operatively, the patient exhibited lower limb weakness and right lower limb dysesthesia. Post-operative 6-month MRI showed complete resolution of the cystic collections, improved spinal cord expansion, and stability of pre-existing myelopathy from D8 to the conus medullaris (Figure 9). Throughout the follow-up period, which was eventually interrupted by the patient, the neurological status did not worsen, and a stable ASIA score of D was registered.

## 4. Discussion

Spinal arachnoid cysts (SACs) account for 1–2% of all spinal tumors [1]. Due to their rarity, most literature consists of case reports and small case series. While secondary SACs are estimated to be less than 10% of the total [12], our study focused solely on secondary cysts to explore their relationship with arachnoiditis and assess clinical outcomes post-surgery.

SACs in adults typically appear between the fifth and sixth decades of life [13,14]. The analyzed cohort of patients were diagnosed between 43 and 68 years, with a mean age of 53.4 years and a typical clinical onset period around the third or fourth decade; a male predominance was highlighted, although there is a notable discrepancy in the literature regarding gender prevalence [3,4,5,6,7,12,13,14].

Although extradurally located SACs are usually more common than intradural ones (10% of all SACs) [2], our series only included intradural cysts. Two patients had syringomyelia; one case (20%) was associated with a spinal cyst, consistent with literature where 33–46% of SAC cases present with syringomyelia. The syringomyelia in one patient extended caudally from the cyst, aligning with Viswanathan et al.’s findings, while the other case had isolated syringomyelia likely due to post-traumatic arachnoiditis, matching literature indicating post-traumatic origins for syringomyelia in 4% of cases [8].

The most common location for SACs is the thoracic spine, with less frequent occurrences in the lumbar, lumbosacral, thoracolumbar, and sacral regions. Cervical involvement is rare [14]. Our study described most cysts in the thoracic spine, with two cases in the dorsolumbar and cervical regions. The higher frequency in the thoracic spine may be due to its length and narrow diameter, which exacerbate neurological symptoms from spinal cord compression [15]. SACs typically span three to four vertebral segments cranio-caudally, and our study’s average was 3.5 levels, similar to Bassiouni et al.’s series [9]. Large cysts, like the one in our study extending over six segments, are not uncommon. Intradural cysts are predominantly dorsal (>75%), although they can also be ventral or intramedullary [8,9,14,15,16,17]. Our cases included dorsal, ventral, and intramedullary cysts.

Clinical presentation of SACs depends on location. For dorsal cysts, back pain is the most frequent symptom, though only half of our patients with posterior cysts reported it. Other common symptoms include radicular pain, paresthesias, muscle weakness, or spastic paralysis [8,15]. In our study, sensory disturbances were present at onset, while transitory motor deficits developed in 40% of cases post-surgery. Cervical cysts typically present with progressive cervical myelopathy, consistent with our findings. Literature suggests ventral cysts often cause muscle weakness and myelopathy, while dorsal cysts are more likely associated with neuropathic pain and paresthesias [15]. In our study, ventral cysts did not predominantly cause motor disturbances at onset, unlike dorsal cysts.

Syringomyelia, causing central spinal cord lesions, is linked to sensory loss, chronic pain, and motor deficits. When it affects the anterior horn, it can lead to arm weakness and absent deep tendon reflexes [17]. In our series of patients with syringomyelia, neither neuropathic pain nor upper limb motor changes were reported. One patient had bowel and bladder issues, likely due to the dorsolumbar syringomyelia.

Diagnosis was confirmed pre-operatively with MRI, which still represents the gold standard. All cysts in our study were associated with myelopathy and edema, showing hypodensity on T1 and hyperdensity on T2, or syringomyelia, consistent with Klekamp et al.’s findings that secondary arachnoid cysts show the abovementioned characteristics due to the arachnoiditis affecting cerebrospinal fluid dynamics [10,18,19].

Surgical treatment involved posterior access via laminectomy for all cases. This approach was used even for cysts spanning more than three vertebral levels, with one case opting for hemilaminectomy, as described by Lee et al. [20]. Minimally invasive techniques, such as laminoplasty suggested by Eroglu et al. [1], are increasingly being described by authors: in our series, we preliminarily applied decompressive laminectomy through piezosurgery in one case as recently published [19]. Laminectomy length did not exceed five segments. Some authors recommend complete resection for cysts up to three segments and partial resection for larger cysts [10]; in our experience, performing complete resection on larger cysts was not associated with complications like post-operative kyphosis [8,9,10,13,14,15,16,17,18,19,20,21,22]. Wang Y. et al. [22] propose complete resection as feasible for small dorsal cysts; in our series, complete resection was applied to all SACs, and recurrences occurred only in a ventral cyst, aligning with higher recurrence rates for anterior cysts. As some authors suggest [15,23,24], for large cysts, microsurgical drainage or fenestration before resection could facilitate wall removal. Aside from one recurrent SAC case, recurrences in our study were associated with myelopathy from ongoing arachnoiditis-related scar formation. Despite meticulous arachnoid dissection and cyst resection, symptoms persisted, supporting literature that surgery alleviates symptoms short-term but does not cure the underlying disease, which is progressive over time [10]. However, surgical treatment stabilized clinical and neurological status for all patients except in one case. The trend of more rapid improvement in motor symptoms versus neuropathic pain, reported in other studies, was confirmed [9,10,15,16,17,18,19]. In cases with syringomyelia, not in line with other series, long-term management with microsurgical dissection and duraplasty was ineffective. One patient required a syringoperitoneal shunt after initial syringopleural shunt failure due to arachnoid adhesions. The efficacy of syringoperitoneal shunting combined with arachnoidolysis in reducing syringomyelia and stabilizing myelopathy supports findings by Mitsuyama et al. and Iwasaki et al. [24,25]. The effectiveness of surgery in achieving lasting clinical improvement depends on patient selection, thorough surgical technique, and ongoing follow-up. Further research with larger patient cohorts and extended follow-up is needed to refine surgical strategies and optimize long-term outcomes for this challenging condition.

Surgical treatment has always been personalized and tailored to the individual patient: in some cases, a hemilaminectomy; in others, a laminectomy; and finally, in a single case, the need for a shunt. Our experience preliminarily demonstrates the importance of personalized medicine: there is no single surgical strategy validated by the literature, but the decision on the therapeutic strategy is taken case by case; the presence of a ventral cyst would seem to be a negative prognostic factor, as it is related to a higher risk of recurrence, while complete resection of the cyst when possible would seem to lower this risk.

Due to their rarity and the presence in the literature of studies limited to small cohorts of patients, there is no consensus on their optimal surgical management. The small number of patients was also the main limitation of this study, along with its retrospective design. Despite the previously mentioned, our preliminary experience could suggest potentially useful work patterns in daily clinical practice and guide future research directions.

## 5. Conclusions

This study demonstrates that surgical resection combined with adhesiolysis can improve clinical outcomes in patients with spinal arachnoid cysts (SACs), potentially relieving neurological symptoms and stabilizing the overall clinical condition. However, careful management and patient selection are essential due to the high risk of complications and recurrences.

## Figures and Tables

**Table 1:** Inclusion and exclusion criteria.

Criteria Type	Criteria
Inclusion	- Adult patients
	- Patients with primary or secondary SACs
	- Patients who underwent surgical treatment
	- Patients with a histopathological diagnosis of SAC
Exclusion	- Patients under 18 years of age
	- Patients with incomplete clinical, radiological, or surgical documentation
	- Patients with spinal cysts incompatible with SAC diagnosis

**Table 2:** Summary of patients’ characteristics, treatment strategies, complications, pre- and post-KPS, pre- and post-ASIA score, and follow-up.

Patient	1	2	3	4	5
Age	46	48	62	43	68
Gender	M	M	M	F	F
Level	T12–L2	C5–C6	T7–T10	T8–T9	T8–T11
Location	Intramedullary	Intradural	Intradural–Extradural	Intramedullary	Intradural
Primary/secondary	Secondary	Secondary	Secondary	Secondary	Secondary
Numbness	Yes	No	Yes	Yes	Yes
Gait disturbances/ataxia	No	Yes	No	Yes	Yes
Lumbar pain	No	No	Yes	Yes	No
Urinary–bowel dysfunction	Yes	No	Yes	No	No
Limb weakness	Yes	Yes	No	No	No
Paraplegia	No	No	Yes	No	No
Sexual disfunction	Yes	No	No	No	No
Surgical treatment	lysis of adherences and syrinx cavity opening	resection of the cystic formation and adhesiolysis	resection of the cystic formation and adhesiolysis	resection of the cystic formation and adhesiolysis	resection of the cystic formation and adhesiolysis
Complications	CSF fistula	infection	None	CSF fistula	None
KPS pre-op (%)	70	50	70	80	90
KPS post-op (%)	80	50	60	90	80
ASIA score pre-op	C	B	B	C	D
ASIA score post-op	D	B	C	C	D
Follow-up (years)	2	2	2	3	1

**Table 3:** Results comparison with main selected studies from the literature.

	Viswanathan et al. [8]	Bassiouni et al. [9]	Klekamp et al. [10]	Eroglu et al. [1]	Mitsuyama et al. [11]
Patients	14	21	21	13	1
Level	C7-L1	D1-D12	C7-L5	T1-T12	T8-T11
Location	Intradural	Intradural	Intradural	Intra and extradural	Intradural
Primary/secondary	primary	complete	primary/secondary	primary/secondary	primary
Surgical treatment	cyst fenestration and partial wall resection through a posterior approach	resection of the cystic formation and adhesiolysis	cyst fenestration and partial wall resection through a posterior approach	resection of the cystic formation and adhesiolysis	Primary micro-dissection, ventriculo-subarachnoid shunt next
Complications	none	infection	CSF fistula	none	none
Neurological status	Improved/stableafter surgery	Improved/stableafter surgery	Improved in primarycysts/worsened insecondary cysts	Improved/stableafter surgery	Improved aftersurgery
Follow-up	6 weeks	3.2 years	10 years	55 months	22 months

## Data Availability

Complete Data is unavailable due to privacy restrictions of Our Institutions.

## Abbreviations

The following abbreviations are used in this manuscript:

MRI	magnetic resonance imaging
CT	computed tomography
NRS	numeric rating scale for pain
